# A DNA methylation based measure outperforms circulating CRP as a marker of chronic inflammation and partly reflects the monocytic response to long‐term inflammatory exposure: A Canadian Longitudinal Study on Aging analysis

**DOI:** 10.1111/acel.13863

**Published:** 2023-05-04

**Authors:** Chris P. Verschoor, Caitlyn Vlasschaert, Michael J. Rauh, Guillaume Paré

**Affiliations:** ^1^ Health Sciences North Research Institute Sudbury Ontario Canada; ^2^ Northern Ontario School of Medicine Sudbury Ontario Canada; ^3^ Department of Health Research Methods, Evidence and Impact McMaster University Hamilton Ontario Canada; ^4^ Department of Medicine Queen's University Kingston Ontario Canada; ^5^ Department of Pathology and Molecular Medicine Queen's University Kingston Ontario Canada

**Keywords:** accelerated aging, chronic inflammation, CLSA, C‐reactive protein, DNA methylation, monocytes

## Abstract

A key hallmark in the age‐related dysfunction of physiological systems is disruption related to the regulation of inflammation, often resulting in a chronic, low‐grade inflammatory state (i.e., inflammaging). In order to understand the causes of overall system decline, methods to quantify the life‐long exposure or damage related to chronic inflammation are critical. Here, we characterize a comprehensive epigenetic inflammation score (EIS) based on DNA methylation loci (CpGs) that are associated with circulating levels of C‐reactive protein (CRP). In a cohort of 1446 older adults, we show that associations to age and health‐related traits such as smoking history, chronic conditions, and established measures of accelerated aging were stronger for EIS than CRP, while the risk of longitudinal outcomes such as outpatient or inpatient visits and increased frailty were relatively similar. To determine whether variation in EIS actually reflects the cellular response to chronic inflammation we exposed THP1 myelo‐monocytic cells to low levels of inflammatory mediators for 14 days, finding that EIS increased in response to both CRP (*p* = 0.011) and TNF (*p* = 0.068). Interestingly, a refined version of EIS based only on those CpGs that changed in vitro was more strongly associated with many of the aforementioned traits as compared to EIS. In conclusion, our study demonstrates that EIS outperforms circulating CRP with regard to its association to health‐traits that are synonymous with chronic inflammation and accelerated aging, and substantiates its potential role as a clinically relevant tool for stratifying patient risk of adverse outcomes prior to treatment or following illness.

## Graphical abstract

The epigenetic inflammatory score (EIS) is based on the weighted sum of DNA methylation levels at 1511 loci across the human genome. It increases with age, obesity, smoking, and chronic inflammatory conditions, and is associated with the likelihood of healthcare utilization and increased frailty over time. EIS also increases significantly in monocyte‐like cells exposed to low‐dose CRP or TNF, suggesting a causal relationship between DNA methylation changes and chronic inflammation in adults.

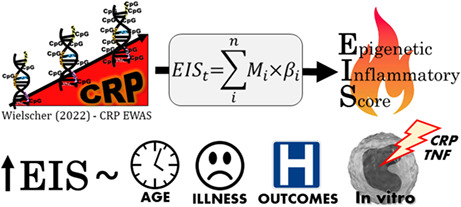

## 1 INTRODUCTION

Aging is related to a loss of physiological integrity, wherein the activity and interaction between multiple key biological systems become dysregulated. A number of factors such as low socioeconomic status, serious illness, and risky behaviors (e.g., smoking) can accelerate this process of dysregulation, promoting vulnerability to injury and disease, while increasing the number of years living with disability (Belsky et al., 2022). Although reducing the prevalence and impact of such harmful factors is fundamental to the process of healthy aging, opportunities lie in the characterization and understanding of the biological systems that change with age and contribute to poor health trajectories in older adults. Recent work has demonstrated that the overall damage or dysregulation of these systems can be quantified using algorithms designed to estimate biological age, risk of death (Ferrucci et al., 2020), pace of aging (Belsky et al., 2022), or overall physiological aberrance (Li et al., 2015) based on the levels of a select battery of cellular or molecular biomarkers. While it is accepted that they are inextricably linked, dysregulation of each individual system does not necessarily occur at the same rate in all individuals (Ahadi et al., 2020); thus, a personalized approach to promote healthy aging may require targeting certain systems in order to benefit the whole.

Of the biological systems that are commonly associated with age‐related outcomes, inflammatory regulation is one of the most well‐established. Inflammation is a conserved mammalian process and in its acute phase, a key response mechanism to danger and damage. Infection or trauma, for example, will elicit a significant release of a variety of pro‐inflammatory molecules, which can activate cells both locally and systemically, leading to the production of inflammatory mediators throughout the body. Under normal circumstances, this process will only last for hours to a few days, and is accompanied by the induction of cells and molecules that promote the resolution of inflammation and wound healing. Dysregulation related to aging disrupts this cycle, promoting an excessive generation of inflammatory mediators, even in the absence of a pathological threat, and resulting in poor resolution of inflammation. The result is a persistent, low‐dose chronic inflammation often referred to as “inflammaging,” which is known to contribute to a number of age‐related outcomes (Fulop et al., 2021). From a population health perspective, the identification and validation of tools that can quantify chronic inflammation over the life‐course would be of value in order to characterize modifiable determinants and target interventions to those at risk of adverse events later in life. However, current approaches involve the quantification of circulating molecular and cellular inflammatory mediators, which do not necessarily reflect prior exposure and commonly suffer from poor reliability for biological (e.g., natural circadian rhythms or genetics) and technical (e.g., assay and laboratory related variability) reasons. Epigenetic‐related measures, which quantify alterations to the genome that are not DNA sequence related, are an attractive alternative to circulating factors as they can persist over long periods of time (Bannister et al., 2022), particularly in hematopoietic cell lineages (Kuribayashi et al., 2021). Previous work from our group (Verschoor et al., 2017) and others (Ligthart et al., 2016; Wielscher et al., 2022) have demonstrated that patterns of cytosine‐guanine (CpG) methylation throughout the human genome are associated with circulating levels of inflammatory biomarkers such as C‐reactive protein (CRP), tumor necrosis factor (TNF), and interleukin (IL)‐6. For CRP, aggregate scores that signify the strength of these patterns in a given individual have been shown to be associated with neurocognitive (Conole et al., 2021; Green et al., 2021; Stevenson et al., 2022) and cardiometabolic traits (Ligthart et al., 2016; Wielscher et al., 2022), and exhibit better test–retest reliability than circulating CRP (Stevenson et al., 2020).

Evidence from the aforementioned studies suggests that a CRP methylation score may be a reliable measure of lifetime exposure to chronic inflammation that would only need a drop of blood to quantify. However, it is unclear the degree to which such an epigenetic measure outperforms traditional biomarkers of inflammation, and whether variation in such a score is mechanistically related to the cellular response to inflammation. In the following study, we generated an epigenetic inflammation score (EIS) based on CpG sites and effect sizes published in a recent large epigenome‐wide association study (EWAS) of circulating CRP from Wielscher et al. (2022). We then compared associations of the EIS and circulating CRP to health‐related traits, biomarkers, and longitudinal outcomes including health‐care utilization, mortality and frailty trajectory in a cohort of 1446 older adults participating in the Canadian Longitudinal Study on Aging (CLSA), and investigated whether EIS increases in monocyte‐like cells exposed long‐term to inflammatory mediators.

## 2 MATERIALS AND METHODS

### 2.1 Analysis of the Canadian Longitudinal Study on Aging dataset

#### 2.1.1 Cohort description

We performed a secondary analysis of the Canadian Longitudinal Study on Aging (CLSA) baseline (2012–2015) and first follow‐up (2015–2018) collection; the CLSA study design and methods have been previously described (Raina et al., 2019). Specifically, it was based on the CLSA comprehensive cohort (baseline dataset version 4.1; follow‐up dataset version 3.0), which includes 30,097 community‐dwelling adults aged 45–86 at recruitment who provided questionnaire data through in‐home interviews and provided additional physical and cognitive assessment data at one of 11 data collection sites nationwide. Within this cohort, a random pool of 10,000 participants was drawn and extensive laboratory measures, including clinical chemistry and hematology, were performed on cryopreserved blood. From this pool, 1479 participants were randomly selected for DNA methylation analysis on their baseline biospecimen. This study was approved by the Health Sciences North Research Ethics Board (#20–030).

#### 2.1.2 DNA methylation analysis in the CLSA

DNA methylation was measured using the Infinium MethylationEPIC BeadChip platform (Illumina) on DNA extracted from peripheral blood mononuclear cells (PBMCs); a summary of this work and the preparation of DNA methylation data can be found at: https://www.clsa‐elcv.ca/doc/3491. Briefly, blood was drawn into CPT vacutainers (BD Biosciences), after which, PBMCs were isolated, resuspended in PBS, and cryopreserved in vapor phase liquid nitrogen. From this, DNA was extracted by QIAsymphony nucleic acid extraction platform using DNA midi kits (Qiagen), and bisulfite‐treated using the EZ DNA methylation kit (Zymo). Measurement of CpG methylation on converted DNA samples by MethylationEPIC array was performed according to manufacturer's recommendations. At each step in this process (i.e., DNA extraction, bisulfite‐conversion and array hybridization and analysis), participant samples were batch‐randomized. After the acquisition of raw data, probe level QC was first performed using functions from the R package “minfi”: the median log intensity of methylated and unmethylated channels were checked using “getQC” and exceeded the recommended threshold of 10.5 for all arrays, while the average probe detection *p*‐value (i.e., methylated and unmethylated signals tested against background) for each array, assessed using the “detection” function, was at least 0.005. Array level QC found that of the 1479 samples initially included for DNA methylation analysis, 4 were removed due to poor bisulfite‐conversion (i.e., <85%), while another 29 were flagged by built‐in outlier detection functions in the R packages “watermelon” and “lumi.” Hence, the final sample included 1446 participants. Missing raw beta values were imputed using a K‐nearest neighbor‐based approach (Lena et al., 2020) and subsequently normalized using inter‐sample (quantile; Hicks & Irizarry, 2015) and intra‐sample (BMIQ; Teschendorff et al., 2013) methods.

#### 2.1.3 Calculation of the epigenetic inflammation score and its derivatives

We calculated the epigenetic inflammation score (EIS) using a weighted‐sum model, ${\mathrm{EIS}}_{t}=\sum_{i}^{n}M_{i}\times \beta_{i}$; here, the EIS for each participant (*t*) represents the summed *M*‐value (M) and effect size (β) product for a given *i*‐th CpG in a panel of n CpGs. The CpGs and related effects sizes were derived from a multi‐ethnic epigenome‐wide meta‐analysis of circulating CRP from 22,774 individuals (Wielscher et al., 2022). Of the 1511 independent loci that the authors identified, 1418 were available to calculate the EIS (Data S1); for this, coefficients from the meta‐analysis (i.e., Effect_metaAnalysis) were employed. After calculation, the EIS was standardized to have a mean of zero and standard deviation of 1. The cEIS represents the EIS calculated using CpG sites and weights from the original set of 1418 that were differentially methylated (i.e., *q*‐value < 0.05) in CRP vs. mock treated THP‐1 cells (see below; Data S2); it was also standardized as above. We also generated 10 additional EIS measures that were based on 68 randomly selected CpGs from the set of 1418 used to calculate EIS (Data S3).

#### 2.1.4 Circulating inflammatory biomarker measures

C‐reactive protein (CRP) and other soluble and cellular biomarkers were measured in blood as part of the baseline collection in the CLSA. C‐reactive protein and albumin were measured in serum using the Cobas 8000 modular analyzer (Roche Diagnostics), TNF and IL‐6 were measured in serum using the Quantikine high sensitivity ELISA (R & D Systems), and absolute measures of monocytes and granulocytes were obtained in whole blood using the Coulter Ac·T diff Analyzer (Beckman Coulter). All measures were log‐transformed and standardized as above.

#### 2.1.5 Demographic and health‐related factors

The following baseline factors were studied in relation to CRP and EIS: age, sex, smoking history, body‐mass index (BMI), inflammatory chronic conditions, frailty, and epigenetic (DNA methylation) age. For all self‐reported variables, refusing or being unable to answer a given question was considered missing.

Smoking history was categorized as never (not smoked 100 cigarettes in their lifetime), former (smoked at least 100 cigarettes, but not in the past 30 days), and current (smoked at least 100 cigarettes and at least one cigarette in the past 30 days). Pack years (PY) exposure was defined as in previous work (Verschoor et al., 2021), and categorized as less than 10 or greater than or equal to 10 PY. Using these two variables, smoking status was defined as: never smoker, former smoker with less than 10 or 10+ PY, and current smoker with less than 10 or 10+ PY.

Body‐mass index was categorized as normal or underweight (BMI < 25), overweight (25 < BMI < 30), or obese (BMI≥30), according to standard World Health Organization definitions; less than 10 individuals were classified as underweight (BMI < 18.5), hence their combination with the normal group.

Chronic conditions that are commonly associated with systemic inflammation included heart disease (including congestive heart failure), peripheral vascular disease, irritable bowel disease, hypertension, myocardial infarction, asthma, chronic lung disease (including chronic obstructive pulmonary disease, chronic bronchitis, and chronic changes to the lung due to smoking), depression, and osteoarthritis (in the knee, hip or hands). All conditions were self‐reported with the exception of depression, which was diagnosed using the 10‐item Center for Epidemiologic Studies Depression Scale (Andresen et al., 1994), where scores ≥10 suggest clinical depression.

Frailty at baseline was estimated using the frailty index approach; specifically, 76 deficits related to chronic conditions, activities of daily living, depression, perceptions of health, satisfaction with life, body mass and social participation, as per previous work (Verschoor et al., 2021). It is calculated as the proportion of deficits present relative to the total sum of deficits considered and ranges from 0 to 1; hence, increasing values represent worse health and greater risk of adverse outcomes. As an example, a person reporting 10 deficits would exhibit a frailty index of 0.131 (i.e., 10 divided by 76). Frailty was defined as missing for any participant missing more than seven deficit variables (~10%). Baseline frailty was considered as both a continuous and categorical variable, the latter being grouped as low (FI <0.1), mild (0.1 < FI <0.2), high (0.2 < FI <0.3), and severe (FI≥0.3).

Four epigenetic measures of biological aging (i.e., epigenetic clocks) were derived at baseline using noob background corrected and normalized beta values (i.e., “preprocessNoob” in the R package “minfi”), as recommended: (Horvath's, 2013) clock (Horvath, 2013), GrimAge (Lu et al., 2019), PhenoAge (Levine et al., 2018), and DunedinPACE (Belsky et al., 2022). DunedinPACE was calculated using the R package “DunedinPACE” (https://github.com/danbelsky/DunedinPACE), while the remaining were calculated using the Horvath DNA methylation age calculator (http://dnamage.genetics.ucla.edu/). For all clocks, standardized delta age values were used, which represent the residual of each respective clock estimate regressed on chronological age, and were transformed to have a mean of zero and standard deviation of 1.

#### 2.1.6 Outcomes

Information regarding prior emergency department visits and hospitalizations were obtained as part of a maintaining contact questionnaire that was administered over the phone between 3.5 and 34.6 months (average, 13.8 months) following the baseline questionnaire. Specifically, participants were asked if they were seen in an emergency department and if they were a patient in a hospital for at least one night anytime in the previous year. Responses were categorized as yes or no. Participant death was confirmed as of July 2019, either by the Ministry or via another source; date of death was not available.

Frailty was assessed at the follow‐up collection for 1320 participants that provided data using the Frailty index approach, as described above; of those that did not provide data, 53 (3.7%) was due to withdrawal from the study and 73 (5.0%) was due to another reason. The 3‐year change was calculated as the difference in frailty from baseline to follow‐up (ΔFI) and categorized relative to a clinically meaningful difference (CMD) of 0.03 (Theou et al., 2020): no change or decrease (ΔFI ≤ 0), or up to 1x CMD (0 < ΔFI <0.03), 2x CMD (0.03 < ΔFI <0.06), or 3x CMD or more increase (ΔFI ≥ 0.09).

### 2.2 Experimental analysis of chronic inflammation

#### 2.2.1 Cell culture and chronic inflammatory exposure

THP‐1 myelo‐monocytic cells (ATCC #TIB‐202) were cultured in suspension in RPMI 1640 media supplemented with 10% fetal bovine serum, 2 mM l‐glutamine, and penicillin–streptomycin. On Day 1, cells were incubated with either PBS (i.e., mock), 1 μg/mL purified CRP (Aviva Systems Biology), 25 pg/mL recombinant TNF (Biolegend), or 25 pg/mL recombinant IL‐6 (Biolegend) at 2.0 × 10^5^ cells/ml in 6‐well plates. On Days 5 and 10, cells were split at 1:4 and resuspended with fresh media and treatment. On Day 14, cells were divided for flow cytometry analysis or DNA/RNA extraction, as below. Each treatment was replicated 4 times in different plates.

#### 2.2.2 Flow cytometry analysis

Approximately 5.0 × 10^5^ cells were pelleted and resuspended in 100 μL staining buffer (PBS, 0.5% bovine serum albumin, 2 mM EDTA). Cells were stained with a 50 μL cocktail containing staining buffer, HLA‐DR‐PE‐Cy7, CCR2‐AmCyan, CX3CR1‐PerCp‐Cy5.5, TLR‐4‐PE, and TLR‐2‐FITC (Biolegend) for 30 mins at room temperature, and washed twice in staining buffer. Cells were resuspended in staining buffer and analyzed using a Beckman Coulter Gallios flow cytometer. Fluorescence is presented as the natural log mean fluorescent intensity (MFI).

#### 2.2.3 Transcriptomic analysis

Cells remaining after flow cytometry staining were lysed and mRNA and DNA extracted using the AllPrep DNA/RNA Mini Kit (Qiagen). Approximately 500 ng was sent to the Centre for Applied Genomics for analysis using the Affymetrix Human Primeview Array. Raw data were normalized by Robust Multichip Average (RMA) using the R package “simpleaffy,” and plate batch effects were removed using the “ComBat” function in the R package “sva”; transcripts below the 95th percentile of fluorescence for all background probes in more than 25% of arrays were removed prior to differential expression analysis. Gene expression differences from the mock treatment (*n* = 4, for each group) were estimated using the “limma” package in R; significant genes were identified as those with an FDR‐adjusted *p*‐value (i.e., *q*‐value) less than 0.05. Pathway analysis for significantly different genes was performed using the Reactome Pathway Browser, version 3.7 (https://reactome.org/PathwayBrowser/).

#### 2.2.4 DNA methylation analysis of THP‐1 cells

Genomic DNA was extracted from THP‐1 cells following inflammatory treatment, as described above. Approximately 1 μg of DNA was sent to the laboratory of Dr. Michael Kobor (University of British Columbia) for methylation analysis. Bisulfite conversion was performed using the EZ DNA methylation kit (Zymo) and measurement of CpG methylation on converted DNA samples using the Illumina Infinium MethylationEPIC array (Illumina) according to manufacturer's recommendations. Raw data were loaded using the “minfi” package in R and background subtraction with dye‐bias normalization, and functional normalization was performed using the function “preprocessFunnorm.” Cross‐hybridizing, sex‐targeting and low detection (i.e., probes having a detection *p* > 0.01 in any single array) probes (*n* = 63,817) were then removed according the published recommendations (McCartney et al., 2016), and plate batch effects adjusted for using ComBat, as above. Global differences in methylation between samples were evaluated by multidimensional scaling (MDS) plot on the top 100,000 most variable probes, and different methylation of *M*‐values relative to mock treatment was performed using “limma,” as described above; probes with an adjusted *p*‐value (i.e., *q*) less than 0.05 were considered differentially methylated. Cellular signatures associated with the top 1000 independent probes identified in the epigenome‐wide association analysis (EWAS) by Wielscher et al. (2022) were obtained using eFORGE v2.0.

### 2.3 Statistical analysis

All continuous variables were summarized as the mean and standard deviation (SD) or 95% confidence interval (CI) and categorical variables as the count and percentage. Bivariate comparisons were tested using Student's *t* test. Associations with chronic conditions were tested using logistic regression, adjusted for age and sex, where the likelihood of having a given condition was presented as the odds ratio (OR) and 95% CI per 1‐SD increase in EIS or CRP. To estimate associations with baseline frailty or the delta age value for each of the epigenetic clocks, linear regression, adjusted for age and sex, was employed. For frailty, coefficients represent the change in standardized EIS or CRP in mild, high or severe category participants relative to low frailty participants, and for the epigenetic clocks, coefficients represent the change relative to a 1‐SD increase in each respective clock. These analyses were repeated for cEIS as well as the set of 10 “random” EIS measures.

Binomial outcomes including emergency department visits, hospitalization and mortality were analyzed using logistic regression. Models were adjusted for age and sex and coefficients represent the OR and 95% CI for a given outcome for every 1‐SD increase in EIS or CRP. Associations with the change in frailty over 3 years were estimated using multinomial regression, adjusting for age, sex and continuous baseline frailty. Coefficients represent the OR and 95% CI for a 1x CMD, 2x CMD, or 3x CMD or more change, relative to no increase, for every 1‐SD increase in EIS or CRP. As above, these analyses were repeated for cEIS as well as the set of 10 “random” EIS measures.

All analyses were performed in R version 4.0.2.

## 3 RESULTS

### 3.1 Characteristics of the epigenetic inflammatory score in older adults

Our study population included 1446 adults aged 45–85 from the Canadian Longitudinal Study on Aging (CLSA). Baseline characteristics can be found in Table S1; briefly, the average age was 63, 51% were female, 10% were current smokers, 32% categorized as obese, 18% were categorized as having high to severe frailty (FI > 0.2) and chronic conditions ranged from 5% for prior myocardial infarction to 40% for hypertension.

**FIGURE 1:**
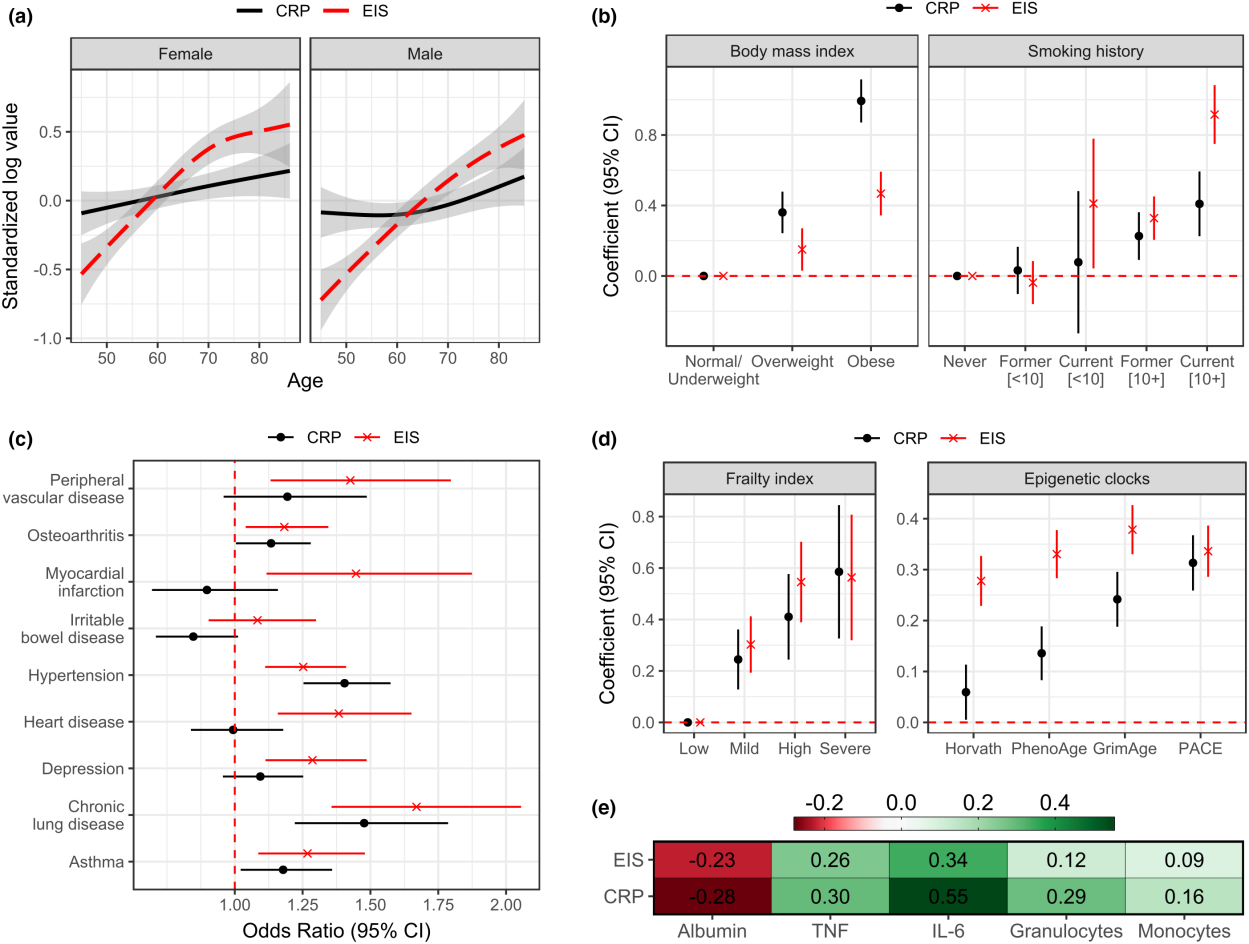
Associations of the EIS and circulating CRP with baseline demographics and health‐related traits in participants of the CLSA (*n* = 1446). (a) Trends in the mean and 95% confidence interval (CI) of the standardized log levels of EIS and CRP are shown for chronological age in women and men. (b) The difference (coefficient) and 95% CI of the standardized measures for each smoking history group (pack‐years shown in square brackets), relative to never smokers, and obesity (BMI) groups, relative to normal/underweight. (c) The odds ratio and 95% CI of having a given inflammatory chronic condition for every 1‐standard deviation (SD) increase in each measure. (d) The difference (coefficient) and 95% CI of the standardized measures for each level of frailty, relative to low frailty, and for every 1‐SD increase in a given epigenetic clock. (e) Pearson correlation coefficients for standardized circulating inflammatory markers relative to EIS or CRP. For b–d, models were adjusted for age and sex, and the red dotted line represents no significant difference or correlation.

Although both EIS and CRP increased with age in both women and men, the slopes for EIS were much steeper, indicating that the difference in average EIS for those in their 70s and 80s relative to those in 40 and 50s was much greater than for CRP (Figure 1a). The correlation (*r*) with age for EIS and CRP was 0.32 and 0.08, respectively (*p* < 0.01), and both tended to be higher in women until the age of 70. With regard to BMI, CRP was found to be relatively higher in the overweight and obese groups, relative to normal/underweight, as compared to EIS (Figure 1b, left). However, the opposite was observed for smoking, as the effect size for EIS was larger than that of CRP for current smokers with less than 10 pack‐years (PY) exposure and anyone with more than 10 PY exposure, relative to never smokers; the difference in effect size for current smokers with more than 10 PY was particularly large (standardized beta (*d*) [95% CI]: EIS = 1.07 [0.90, 1.23], CRP = 0.41 [0.23, 0.59]; Figure 1b, right). Increasing EIS was strongly associated with inflammatory conditions such as peripheral vascular disease (standardized OR [95% CI] = 1.43 [1.13, 1.80]), myocardial infarction (1.45 [1.12, 1.87]), heart disease (1.38 [1.16, 1.65]), depression (1.29 [1.11, 1.49]) and chronic lung disease (1.67 [1.36, 2.06]), substantially more so than CRP, with the exception of hypertension, where the estimate for CRP was stronger (Figure 1c). This discrepant association between hypertension and CRP was strongly confounded by obesity, as the difference in effect size between EIS and CRP mostly disappeared after adjusting for participant BMI (CRP: 1.16 [1.02, 1.31]; EIS: 1.12 [0.99, 1.27]). Although the effect sizes related to baseline frailty were largely similar between the EIS and CRP (Figure 1d, left), the correlations for EIS with the Horvath, PhenoAge and GrimAge epigenetic clocks were markedly larger than that of CRP (*d* = 0.14–0.38; Figure 1d, right). Finally, we performed a correlation analysis for EIS and CRP with circulating levels of molecular and cellular biomarkers commonly associated with both acute and chronic inflammation. Unlike the previous analyses of health‐related traits, correlations were consistently stronger for these biomarkers and CRP, particularly IL‐6 and myeloid cell counts (Figure 1e).

### 3.2 Associations of EIS and circulating CRP with longitudinal health outcomes

**FIGURE 2:**
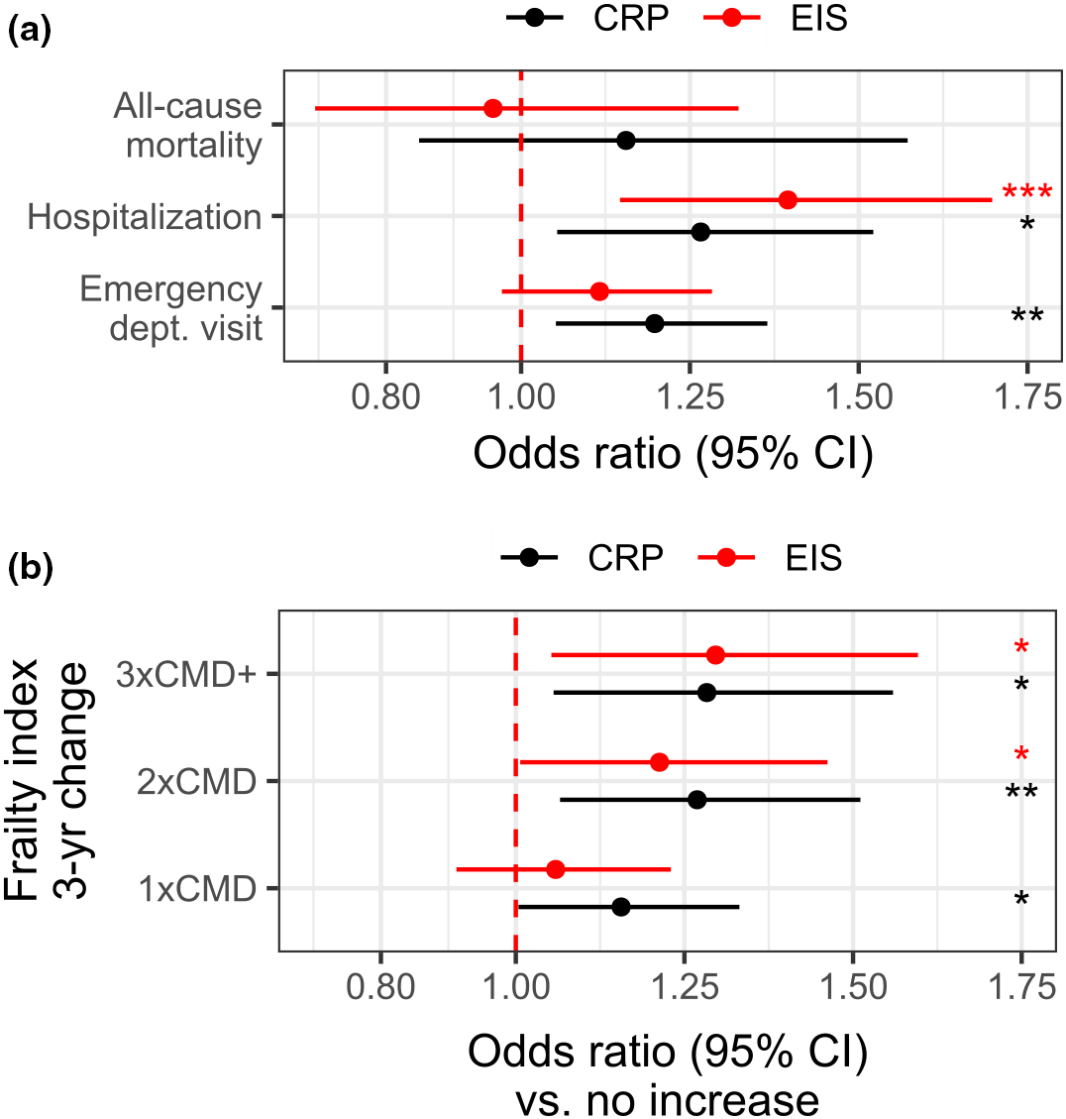
Associations of EIS and circulating CRP with longitudinal health outcomes. (a) The odds ratio (OR) and 95% confidence interval (CI) of having a emergency department visit, overnight hospitalization or death for each standardized measure. (b) Relative to having no increase in frailty at 3‐year follow‐up, the OR and 95% CI of having an increase in frailty that was up to 1x, 2x, and 3x or more than the clinically meaningful difference (CMD) for each measure. All models were adjusted for age and sex (and baseline frailty in b), and nominal *p*‐values denoted as follows: ***p < 0.001; **p < 0.01; *p < 0.05. ORs in (a, b) are relative to a 1‐SD increase in each measure.

To investigate whether EIS is a sensitive predictor of long‐term health in older adults, we estimated its association to health‐care utilization, all‐cause mortality and the change in frailty over 3 years (Table 1). As shown in Figure 2a, the likelihood of emergency department use was significantly associated with a 1‐SD increase in CRP (standardized OR [95% CI] = 1.20 [1.05, 1.36]), but not EIS (1.12 [0.97, 1.28]). Both were significantly associated with overnight hospitalization, more so for EIS (1.40 [1.15, 1.70]) than CRP (1.27 [1.05, 1.52]). Neither of the measures were associated with all‐cause mortality.

**TABLE 1:** Summary of longitudinal outcomes for CLSA participants.

	Total
	(*n* = 1446)
*Emergency dept. visit*	
No	1082 (74.8%)
Yes	288 (19.9%)
Missing	76 (5.3%)
*Hospitalization*	
No	1244 (86.0%)
Yes	127 (8.8%)
Missing	75 (5.2%)
*All‐cause mortality at 3 years*	
Alive	1402 (97.0%)
Deceased	44 (3.0%)
*ΔFrailty at 3 years (continuous)*	0.00631 (0.044)
Missing	182 (12.6%)
*ΔFrailty at 3 years (categorical)*	
None	582 (40.2%)
1xCMD	365 (25.2%)
2xCMD	182 (12.6%)
3xCMD+	135 (9.3%)
Missing	182 (12.6%)

*Note*: Continuous data summarized as the mean (standard deviation) and categorical as the count (frequency). The change (Δ) in frailty at 3‐year follow‐up was categorized relative to clinically meaningful differences (CMD; ~0.03).

Using multinomial regression, we estimated the likelihood of having an increase in the frailty index after 3 years at intervals relative to the commonly employed clinically meaningful difference (CMD): up to 1x CMD (0 < ΔFI <0.03), 2x CMD (0.03 < ΔFI <0.06), or 3x CMD or more (ΔFI ≥ 0.06; Figure 2b). The likelihood of having an increase in frailty followed a similar dose–response pattern for both EIS and CRP, and differed little. For every 1‐SD increase in EIS, the odds of having a 1x, 2x or 3x CMD or more change in frailty increased 1.06 (95% CI = 0.91, 1.23), 1.21 (1.01, 1.46) and 1.30 (1.05, 1.60) fold, respectively; for CRP, the odds increased 1.16 (1.00, 1.33), 1.27 (1.07, 1.51) and 1.28 (1.06, 1.56) fold, respectively. Interestingly, when modeled together, EIS and CRP exhibited associations of similar magnitude and significance with a 3x CMD or greater change in frailty (EIS = 1.24 [1.00, 1.53], CRP = 1.24 [1.01, 1.51]).

### 3.3 Investigating the epigenetic response to long‐term inflammatory exposure in vitro

**FIGURE 3:**
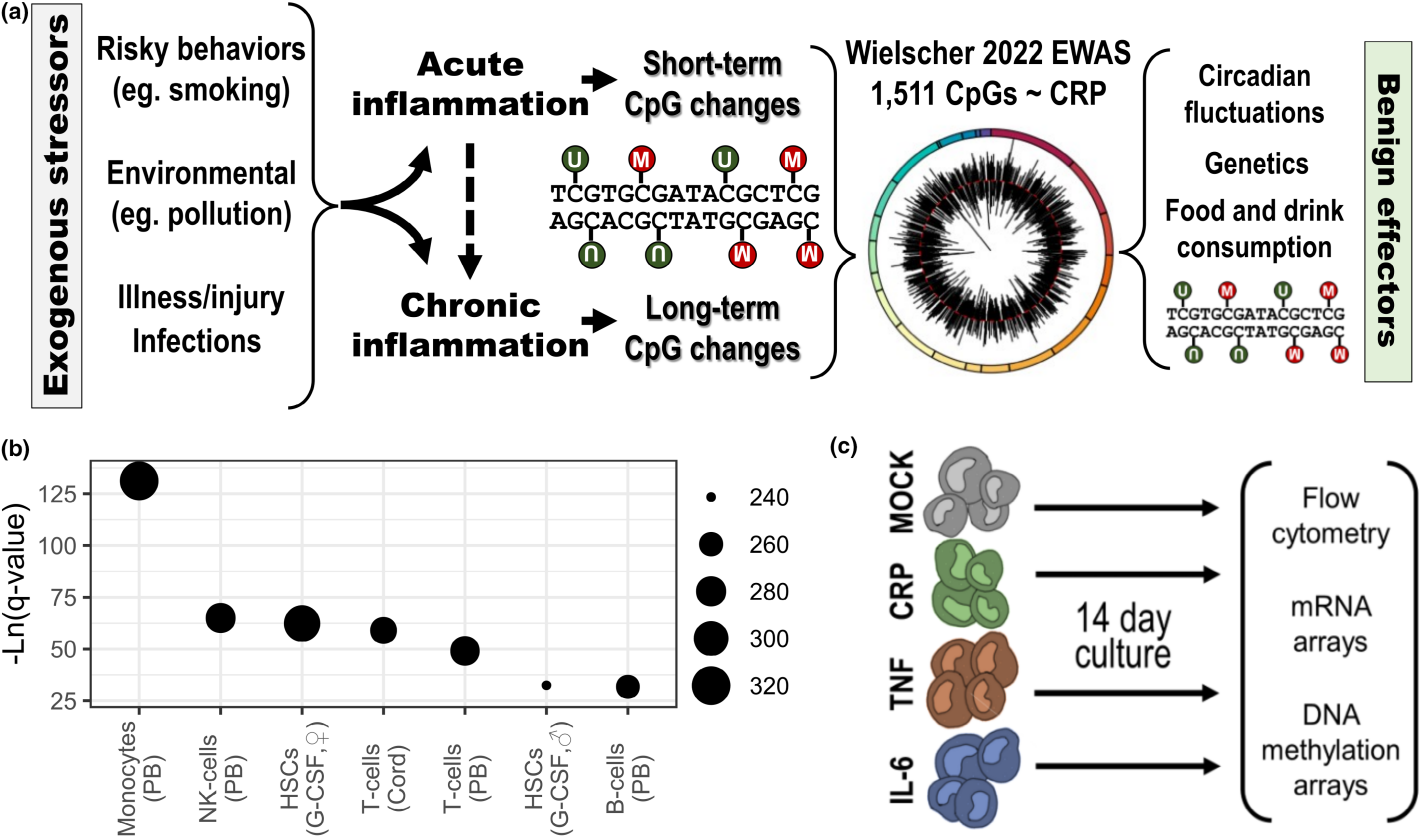
Overview of the rationale to study chronic inflammatory exposure in THP‐1 myelo‐monocytic cells. (a) A conceptual model describing the nature of DNA methylation sites that are associated with the inflammatory biomarker C‐reactive protein (CRP), implicating both exogenous stressors and benign effectors. (b) Results from the eFORGE v2.0 cell signature analysis of the top 1000 CpG sites identified in the 2022 EWAS by Wielscher and colleagues; the prediction of different blood cell types [peripheral blood (PB) or cord blood leukocytes and female/male hematopoietic stem cells (HSCs) following GM‐CSF therapy] is based on FDR‐adjusted *p*‐value (i.e., *q*‐value) and the number of classified probes. (c) The design of the long‐term THP‐1 inflammatory exposure experiment.

Due to the multitude of factors that can influence DNA methylation levels, it is unclear whether variation in EIS actually represents an allostatic response to inflammation (Figure 3a). To investigate this, we used a myelo‐monocytic THP‐1 cell culture model where cells were exposed to physiologically relevant, low‐dose concentrations of CRP, TNF or IL‐6. THP‐1 cells were employed because the complex cellular signature of CpG sites identified by Wielscher et al. (2022) indicated a predominant contribution by peripheral blood monocytes (Figure 3b), and they are easily maintained in suspension, while sharing many other phenotypic and functional properties of peripheral blood monocytes. Cells (*n* = 4, per treatment) were exposed to either PBS (mock) or CRP (1 μg/mL), TNF (25 pg/mL) or IL‐6 (25 pg/mL) for 14 days, after which the intracellular and extracellular phenotype was analyzed (Figure 3c).

**FIGURE 4:**
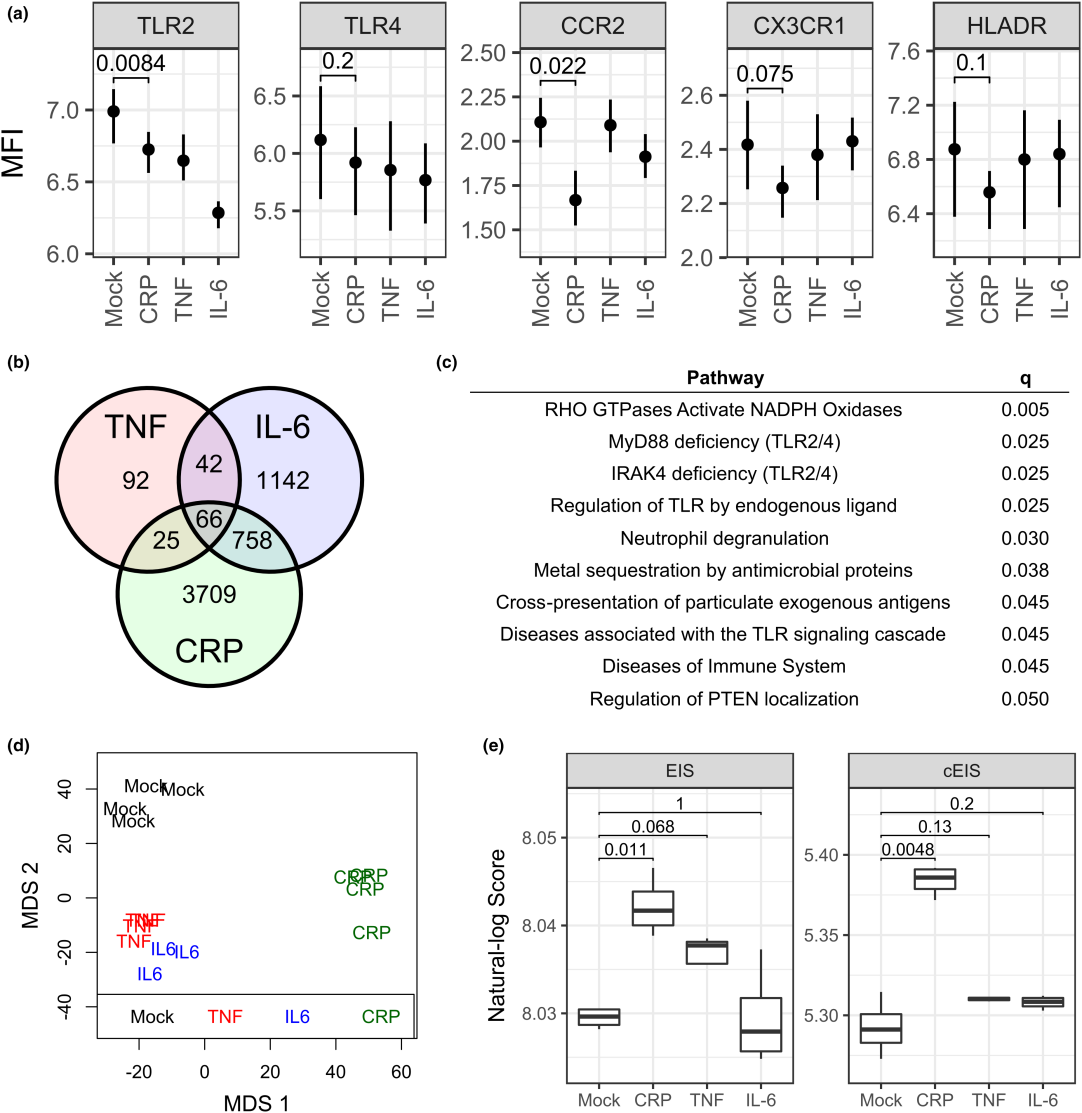
Molecular alterations in THP‐1 myelo‐monocytes as a result of long‐term exposure to CRP, TNF and IL‐6. (a) Cell‐surface receptor expression following 14‐day treatment, expressed as the log mean fluorescent intensity (MFI); *p*‐values shown for CRP vs. mock comparison. (b) Venn diagram of mRNA identified as differentially expressed relative to mock treatment following transcriptomic analysis. (c) Reactome pathways enriched in the differentially expressed mRNA set for CRP vs. mock; only those with *q* ≤ 0.05 are shown. (d) Clustering of replicates according to treatment is shown by multidimensional scaling (MDS) plot for the top 100,000 most variable CpGs across all samples. (e) The EIS and cEIS in response to each treatment; *p*‐values for each comparison are shown.

After long‐term exposure, all inflammatory treatments induced a reduction in cell‐surface expression of immune receptors TLR‐2, TLR‐4, CCR2, CX3CR1, and HLA‐DR, although to different degrees depending on the treatment and receptor (Figure 4a); the reduction was pronounced for CRP, especially for TLR‐2 (*p* = 0.008), CCR2 (*p* = 0.022), and CX3CR1 (*p* = 0.075). Transcriptomic analysis using the Affymetrix Primeview microarray indicated that CRP treatment caused the differential expression (*n* = 4558, *q* < 0.05; Data S4) of substantially more genes than TNF (*n* = 225; Data S5) or IL‐6 (*n* = 2008; Data S6), although ~40% of genes differentially expressed by TNF or IL‐6 were shared with that of CRP (Figure 4b). Genes differentially expressed by CRP were observed to be predominantly involved in immune related pathways, especially TLR signaling, antigen presentation, and the regulation of oxidative stress (Figure 4c); neither TNF or IL‐6 were enriched for pathways at *q* < 0.05.

To quantify changes to genome‐wide DNA methylation levels following long‐term inflammatory treatment, we used the Illumina MethylationEPIC array. Cells treated with CRP clustered separately from other treatments, although the distinction from TNF and IL‐6 was mostly apparent on the second multidimensional scaling (MDS) axis (Figure 4d). Similar to the transcriptomic analysis, substantially more CpG sites were differentially methylated in response to CRP (*n* = 32,357, *q* < 0.05; Data S7) as compared to TNF (*n* = 3465; Data S8) or IL‐6 (*n* = 3305; Data S9), although there were not as many shared significant loci between the CRP treatment and that of TNF (*n* = 1163, 34%) or IL‐6 (*n* = 452, 14%); only a small number of the sites differentially methylated by CRP were shared with those that compose EIS (*n* = 68, ~0.2%; Data S2). Using the weighted‐sum approach as described above, we calculated EIS and found that it was significantly greater following CRP treatment as compared to mock (*p* = 0.011), and also higher after TNF treatment, although not significant (*p* = 0.068; Figure 4e, right). Based on our hypothesis that not all CpGs identified by Wielscher et al. (2022) are mechanistically related to the cellular response to inflammation, we also calculated a refined version of EIS, called chronic EIS (cEIS), which employs only those 68 CpGs that were commonly identified in the Wielscher EWAS and by our CRP differential methylation analysis (Data S7). As expected, the difference between mock and CRP treated cells was even greater for cEIS (*p* = 0.005); while greater for TNF and IL‐6 relative to mock, it was not significant (*p* > 0.10; Figure 4e, left).

### 3.4 Associations of a refined epigenetic inflammatory score with participants factors and health‐traits

**FIGURE 5:**
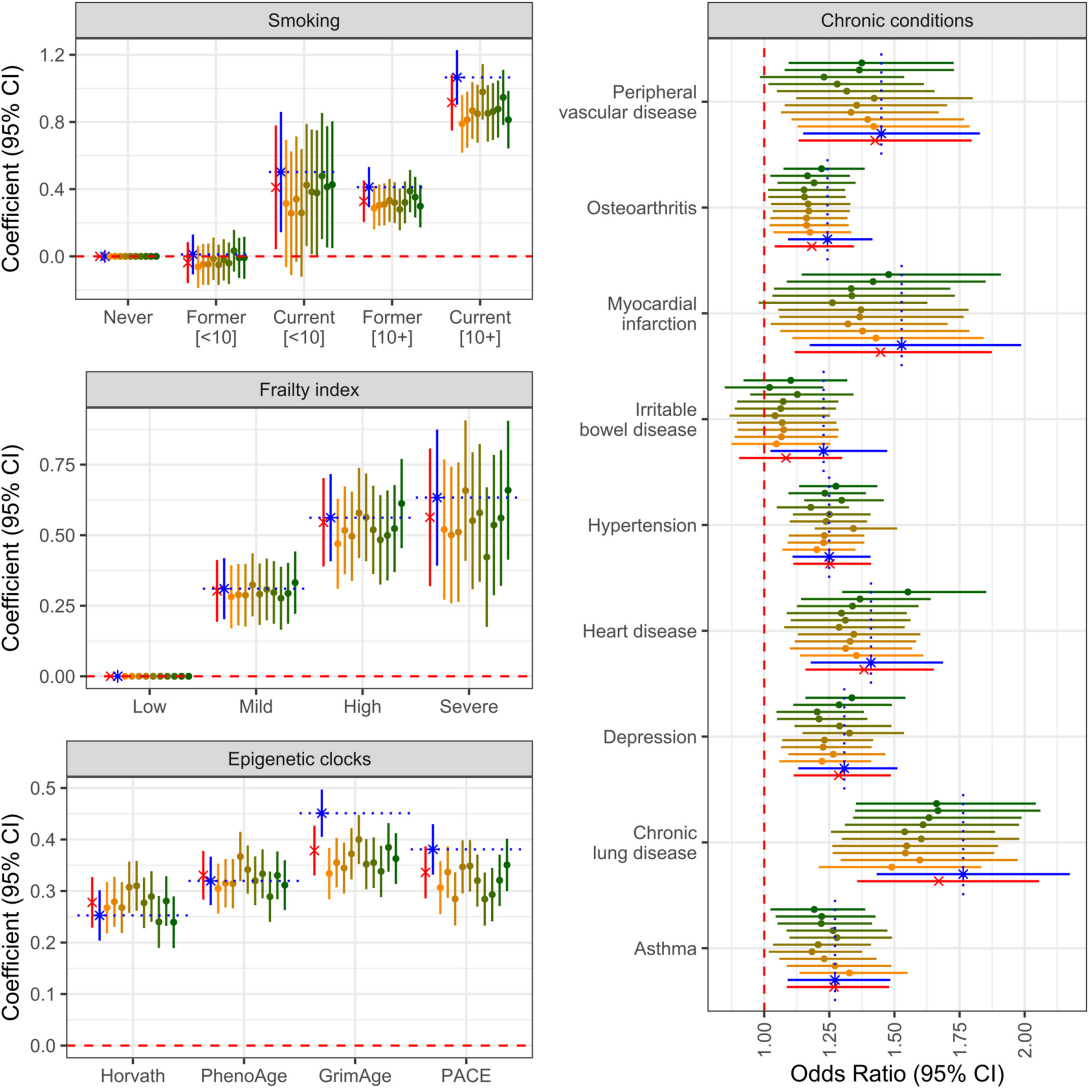
Associations of participant factors and health traits with EIS, cEIS and a random set of epigenetic scores. Effect size estimates and 95% confidence intervals are shown for EIS (red x), cEIS (blue asterisk), and 10 epigenetic scores (yellow to green dots), which represent a random set of 68 CpGs from the 1418 CpG list that was used to calculate EIS. cEIS was calculated using the 68 CpGs that were shared between Wielscher's EWAS set and those differentially methylated in THP‐1 cells stimulated with CRP. The red dotted line indicates no significance at the nominal level, while the blue dotted line represents the estimate for cEIS within a given variable category.

Due to its enrichment for CpG sites that reflect the cellular response to long‐term CRP exposure, we hypothesized that cEIS would exhibit stronger associations to participant factors than EIS, particularly those for which EIS outperformed CRP. To test this, we compared cross‐sectional associations with smoking, frailty, epigenetic clocks, and chronic conditions among EIS, cEIS, and 10 EIS instruments calculated using random selections of 68 CpGs from the set that composes EIS (Data S3). As shown in Figure 5, cEIS appeared to exhibit stronger associations than EIS or the random set with smoking history, chronic lung disease, osteoarthritis, irritable bowel syndrome and the epigenetic clocks GrimAge and PACE, although in most cases there was significant overlap in error. In contrast, correlations with factors such as depression, peripheral vascular disease, and the Horvath and PhenoAge epigenetic clock, for which EIS exhibited stronger associations than CRP, were relatively similar among cEIS, EIS, and the random set.

## 4 DISCUSSION

The overarching goals of this study were to demonstrate that EIS is a superior measure of chronic inflammation as compared to circulating CRP, while providing evidence that at least a portion of the CpGs previously identified by Wielscher et al. (2022) are directly related to the cellular response to CRP as a result of chronic inflammation. Although some factors and longitudinal outcomes were not associated more strongly with EIS as compared to CRP, many that are synonymous with chronic inflammation and accelerated aging were, thereby supporting our hypothesis. Importantly, using an in vitro model of long‐term inflammatory exposure, we showed that EIS increases in response to CRP, even though only a small fraction of CpGs used to calculate EIS were found to be differentially methylated by CRP treatment. A refined score based only on these shared sites (i.e., cEIS) actually exhibited stronger associations with some participant factors and health‐traits as compared to EIS, suggesting that signatures based on the cellular response to inflammation may be more efficacious; however, the underlying pathological mechanisms remains undefined.

Although recent studies have shown that epigenetically derived measures of inflammation are more strongly associated with measures of cognitive function (Conole et al., 2021) and brain structure (Conole et al., 2021; Green et al., 2021) as compared to circulating biomarkers, a comprehensive analysis of participant factors and health‐related traits has yet to be performed. Our data indicate that EIS was associated more strongly with age, smoking history, most chronic conditions, and epigenetic clock metrics as compared to CRP. The trends with increasing age were particularly interesting, where EIS was more linear than CRP at younger ages. This suggests that EIS may prove to be especially useful for detecting exposure to chronic inflammation in younger adults. For the epigenetic clock measures, associations were markedly larger for the mortality‐risk clocks PhenoAge and GrimAge, even though EIS shares a very small number of sites with these measures; less than 2% (*n* = 9) of EIS CpGs are used to calculate PhenoAge and no more than 8 CpGs were included in GrimAge (this is only an estimate based on the probe list provided with the Horvath online calculator since the GrimAge CpG list has not been published). In contrast to the aforementioned traits, CRP did exhibit stronger correlations to other circulating inflammatory markers, especially so for IL‐6, monocytes, and granulocytes. These observations were in line with our hypothesis, as circulating factors related to inflammation and oxidative stress are known to fluctuate similarly in the short‐term, and has been shown in studies of exercise (Kasapis & Thompson, 2005) or ambient temperature (Wu et al., 2017). Although there were no obvious differences in the associations for EIS and CRP with the longitudinal outcomes investigated, we did find evidence of independence; specifically, both measures were significantly associated with a 3x or greater CMD increase in frailty over 3 years when they were modeled together. This suggests that while circulating inflammatory markers such as CRP may not be as useful a measure of chronic inflammation as those based on DNA methylation, they nonetheless can offer unique predictive utility for longitudinal outcomes, possibly as a complimentary measure specific for acute inflammation.

Part of our hypothesis regarding the superiority of EIS over CRP as a measure of chronic inflammation lies in the nature of the measure itself. As it is based on the degree of variability in DNA methylation patterns of white blood cells, it can theoretically accumulate over time depending on the level of exposure (Jones et al., 2015), and hence, should better reflect the long‐term allostatic response of cells to inflammation. Circulating factors such as CRP are known to exhibit minor and seemingly benign fluctuations on a day‐to‐day basis (Halonen et al., 2010; Nathan et al., 2021), and can even be maintained at chronically high levels due to genetic predisposition without the apparent incidence of associated diseases, as has been demonstrated in the Indigenous Tsimane population (Freese et al., 2018; Vasunilashorn et al., 2011). CRP also exists as multiple, pathologically relevant structural isoforms that are not easily measured using a single assay and do not necessarily correlate with one another (Melnikov et al., 2022). To support our hypothesis that EIS at least partly reflects the cellular response to chronic inflammation, we used an in vitro model of long‐term, low‐dose inflammatory exposure where the response of THP‐1 myelo‐monocytic cells to physiological relevant levels of CRP was compared to a mock control. We chose the THP‐1 cell line because the CpG signature on which EIS is based suggested that the variance in DNA methylation levels could best be attributed to blood monocytes, which is not surprising since monocytes are well‐known for their sensitivity to a variety of inflammatory signals (Shi & Pamer, 2011). Following treatment, cells exhibited alterations consistent to what we and others have shown for monocytes in the context of aging, including reduced surface expression of TLRs, CCR2 and CX3CR1 (Seidler et al., 2010; Verschoor et al., 2014) and the differential expression of genes belonging to immune‐related pathways, particularly TLR‐signaling (van Duin et al., 2007). Approximately 40% of differentially expressed genes identified following TNF and IL‐6 treatment were shared with CRP, which is not surprising, as the endpoint of CRP signaling, NF‐kB and MAPK pathway activation (Fernández et al., 2002) via Fc‐gamma receptor I (CD64) and II (CD32; Bharadwaj et al., 1999), is similar to that of TNF and IL‐6, which signal through their own family of receptors (Gane et al., 2016; Schett, 2018).

Although the effect of CRP on global DNA methylation patterns was substantial, resulting in over 32,000 CpG sites being found significantly altered, relatively few (*n* = 68) of these sites were shared with those identified by Wielscher et al. (2022). Nonetheless, EIS was observed to be significantly elevated by CRP treatment, and was higher following TNF treatment, albeit only at *p* < 0.10. Interestingly, a refined version of EIS based only on those shared sites, cEIS, exhibited stronger associations to a number of inflammatory‐related factors and health‐traits relative to EIS, beyond what is likely to be random chance. While our findings are not conclusive, they strongly suggest that elements of EIS do reflect the cellular response to inflammation and that such responsive CpG sites may better serve future epigenetic measures of chronic inflammation. These sites may also represent attractive targets of intervention for individuals suffering from immune dysfunction as a result of chronic inflammation, although the molecular mechanisms of inflammatory‐related DNA methylation alterations would need to be better characterized. Previous work using THP‐1 cells and primary monocytes has demonstrated that chronic TNF treatment induces a form of “transcriptional memory,” which leads to greatly enhanced NF‐kB‐mediated gene expression following re‐treatment (Zhao et al., 2020). This is similar to an epigenetic phenomenon known as trained immunity, which can be induced by both LPS and glucose exposure, and importantly, relies on the activity of the transcription factor CTCF (de Laval et al., 2020; Edgar et al., 2021), for which EIS CpGs are significantly enriched in binding sites (Wielscher et al., 2022). Increasing signal transduction through NF‐kB and/or via CTCF would provide a mechanistic basis for DNA methylation to act as a driver of chronic inflammation in older adults in a positive feedback fashion. Although this needs to be verified experimentally, it may underlie previous findings of age and age‐related inflammation promoting an inflammatory state in monocytes and myeloid‐lineage cells as well as systemically (Puchta et al., 2016).

In summary, our study provides strong evidence that DNA methylation‐based measures are excellent proxies to quantify exposure to chronic inflammation. As proof‐of‐principle, we further showed that those CpG sites related to the cellular response to low‐dose inflammatory stimuli may be the most influential in determining the utility of those measures. However, there are two important caveats to this work that should be considered moving forward. First, it is unlikely that the complexity of chronic inflammation, which has been implicated to involve a variety of classes of mediators (e.g., protein, cellular, metabolic, and nucleic), is fully captured via a methylation‐based proxy of circulating CRP alone. In fact, some would argue that CRP best serves as marker of acute inflammation, while other measures better reflect the chronic state, particularly age‐related chronic inflammation; this is evident in the CLSA, as CRP correlates with age much weaker than TNF or IL‐6 (*r*: CRP = 0.08; TNF = 0.32, IL‐6 = 0.27). In this light, a more complete epigenetic measure of inflammation would likely reflect multiple measures of chronic inflammation or even a composite score of soluble measures such as inflammatory age (iAge), which is strongly influenced by levels of inflammatory‐related chemokines (e.g., CXCL9, CCL11, and CCL3) and cytokines (e.g., IL‐1β, IFN‐α, and IL‐5) and is positively correlated STAT‐phosphorylation in monocytes following stimulation (Sayed et al., 2021). Secondly, the cellular response to chronic inflammation may involve multiple cell types, perhaps including natural‐killer (NK) cells, the phenotype of which we recently showed to be significantly associated with circulating IL‐6 and TNF (Picard et al., 2022). This is particularly important given the utility of deriving epigenetic scores from blood, which represents a mixture of cellular populations, each with a potentially different propensity to manifest chronic inflammation via DNA methylation, as suggested by our eFORGE cell signature analysis. Hence, additional investigation into the response of individual cell populations to chronic inflammation and how this is reflected at the level of the methylome will be important to improve both the performance of epigenetic inflammatory measures and our understanding of the causal pathway on which it is based. While immortalized cells may be convenient models to do so, future work into the cellular response to inflammation should focus on changes that occur in primary cells.

## AUTHOR CONTRIBUTIONS

CPV obtained funding for the study, conceived the study design, performed laboratory work and analyses, and prepared the manuscript. CV, MJR, and GP provided feedback on the study design, analysis, and interpretation and contributed to the manuscript.

## CONFLICT OF INTEREST STATEMENT

The authors have no conflicts of interest to declare.

## Supporting information


Table S1:

Data S1:

## Data Availability

Investigators wishing to access data from the CLSA can obtain more information from its website (www.clsa‐elcv.ca). All other data can be requested from the corresponding author upon reasonable request.
