# Supplementary material for: A DNA methylation based measure outperforms circulating CRP as a marker of chronic inflammation and partly reflects the monocytic response to long‐term inflammatory exposure: A Canadian Longitudinal Study on Aging analysis
Source: Aging Cell. 2023 May 4;22(7):e13863. doi: 10.1111/acel.13863 (PMC10352553; doi:10.1111/acel.13863)
Supplement: Supplementary file 1 — Table S1: [file ACEL-22-e13863-s002.docx]

# Supplemental Tables

**Supplemental Table 1:** Summary of baseline demographic and health-related variables of older adults.

|  | **N=1446** |
| --- | --- |
| **Age** | 63 (10.3) |
| **Sex** |  |
| Female | 732 (50.6%) |
| Male | 714 (49.4%) |
| **Smoking history** |  |
| Never | 653 (45.2%) |
| Former [<10 PY] | 312 (21.6%) |
| Current [<10 PY] | 24 (1.7%) |
| Former [10+ PY] | 319 (22.1%) |
| Current [10+ PY] | 137 (9.5%) |
| Missing | 1 (0.1%) |
| **Body-mass index (BMI)** |  |
| Normal/Underweight | 402 (27.8%) |
| Overweight | 572 (39.6%) |
| Obese | 469 (32.4%) |
| Missing | 3 (0.2%) |
| **Frailty index** |  |
| Low (0<FI<0.1) | 475 (32.8%) |
| Mild (0.1<FI<0.2) | 703 (48.6%) |
| High (0.2<FI<0.3) | 201 (13.9%) |
| Severe (FI≥0.3) | 64 (4.4%) |
| Missing | 3 (0.2%) |
| **Heart disease** |  |
| Yes | 174 (12.0%) |
| Missing | 5 (0.3%) |
| **Peripheral vascular disease** |  |
| Yes | 86 (5.9%) |
| Missing | 5 (0.3%) |
| **Irritable bowel disease** |  |
| Yes | 143 (9.9%) |
| Missing | 3 (0.2%) |
| **Hypertension** |  |
| Yes | 571 (39.5%) |
| Missing | 6 (0.4%) |
| **Myocardial infarction** |  |
| Yes | 71 (4.9%) |
| Missing | 5 (0.3%) |
| **Asthma** |  |
| Yes | 216 (14.9%) |
| Missing | 4 (0.3%) |
| **Chronic lung disease** |  |
| Yes | 109 (7.5%) |
| Missing | 7 (0.5%) |
| **Depression** |  |
| Yes | 253 (17.5%) |
| Missing | 5 (0.3%) |
| **Osteoarthritis** |  |
| Yes | 389 (26.9%) |

Continuous data summarized as the mean (standard deviation), and categorical as count (frequency). PY, pack-years.
